# Psychology, stress, insomnia, and resilience of medical staff in China during the COVID-19 policy opening: a cross-sectional survey

**DOI:** 10.3389/fpubh.2023.1249255

**Published:** 2023-08-25

**Authors:** Zhen Cheng, Yuanling Tao, Ting Liu, Siyue He, Yu Chen, Li Sun, Zongtao Chen

**Affiliations:** Health Management Centre, First Affiliated Hospital of Army Medical University, Chongqing, China

**Keywords:** COVID-19, medical staff, psychology, stress, insomnia, resilience

## Abstract

**Background:**

Since 8 January 2023 China has liberalized its control of COVID-19. In a short period of time, the infection rate of COVID-19 in China has risen rapidly, which has brought a heavy burden to medical staff. This study aimed to investigate the psychological status, stress, insomnia, effort-reward imbalance, resilience, and influencing factors of medical staff in China during the period of epidemic policy liberalization.

**Methods:**

This survey was conducted from 6 February to 27 March 2023 with non-random sampling. An online questionnaire survey was conducted using HADS, PSS-14, ISI, ERI, and the resilience assessment scale for medical staff. The levels of psychological, stress, insomnia, effort-reward imbalance, and resilience of medical staff during the pandemic policy opening period were measured.

**Results:**

A total of 2,038 valid questionnaires were collected. 68.5% and 53.9% of medical staff had different degrees of anxiety and depression, respectively. Excessive stress, insomnia, and high effort and low reward were 40.2%, 43.2%, and 14.2%, respectively. Gender, Profession, education level, and age are important factors that lead to anxiety and depression. Women, nurses, higher education, longer working years and hours, high effort, and low reward are risk factors for the above conditions. There was a certain correlation among the five scales, among which anxiety, depression, stress, insomnia, effort-reward imbalance, and other factors were positively correlated, while resilience was negatively correlated with these factors.

**Conclusion:**

This study found that anxiety, depression, stress, insomnia, and other psychological problems of medical staff in China during the policy opening period of COVID-19 were more serious than before. At the individual and organizational levels, it is necessary to improve the well-being of medical staff, optimize the allocation of human resources, and promote the mental health of medical staff with a focus on prevention and mitigation, with the entry point of improving resilience and preventing the effort-reward imbalance.

## 1. Introduction

In recent years, frequent occurrences of public health emergencies, especially sudden major infectious diseases, have posed a huge threat and impact on the medical and health system (1). By March 2023, 676 million people had been infected with COVID-19, including 6.88 million deaths (2). For nearly 3 years, from December 2019 to 2022, China adopted the “dynamic zero COVID-19” strategy to deal with this complex epidemic. In view of the differences between the Omicron variant of COVID-19 and the early SARS-CoV-2 prototype strain and other variants (3), its clinical manifestations are significantly shortened incubation period, significantly increased spread rate, asymptomatic and mild patients accounting for the vast majority, and significantly reduced pathogenicity and fatality rate (4). Since 8 January 2023, China has significantly adjusted its epidemic prevention policy against COVID-19 (5). No more quarantine measures would be imposed on those infected with COVID-19, no more close contacts would be identified, and high-or low-risk areas would no longer be divided. Graded and categorical admission and treatment of people infected with the virus and adjusted medical security policies would be implemented in a timely manner. The detection policy was adjusted to “Voluntary inspection.”

Within a short period of time after the major policy adjustment, China’s COVID-19 infection rate is significantly higher than in previous periods. Due to the huge population base and the problem of an aging population (6, 7), COVID-19 patients with severe disease, mainly the older adult, have brought a heavy burden to medical staff. In the face of this large-scale infectious public health event, the medical staff are under great pressure due to high work intensity, high risk of infection, uncertain medical technology, and personal capacity. Previous studies have shown that persistent stressful events can lead to anxiety, depression symptoms, insomnia, and other mental health problems for medical staff. Previous surveys during the pandemic found that 36.9% of medical staff in Wuhan, China, had subthreshold mental health disorders (8). The levels of depression and anxiety among medical staff in Shanghai were significantly higher than the norm (9). Medical staff had higher levels of anxiety (13.1%/7.6%) and depression (24.7%/16.0%) than non-medical staff (10), and 25% of medical staff reported poor sleep quality (11). At least 20% of medical staff have been diagnosed with post-traumatic stress disorder (12), and some medical staff also suffer from empathy fatigue and burnout (13, 14). The anxiety, depression, insomnia, and other conditions of medical staff will further affect and change their psycho-emotional and behavioral patterns, including job dissatisfaction, reduced professional efficiency and personal achievement, chronic fatigue, sleep disorders, negative emotional states (e.g., anxiety and depression), and gastrointestinal problems (15). If they go without timely psychological support and intervention, it will not only affect the mental health of medical staff, reduce the quality of medical services, and threaten medical safety, but also affect the prevention and control of the epidemic (11, 16). Therefore, the investigation and improvement of the psychological health of the clinical first-tier medical staff during the period is very important.

Resilience is the ability to help medical staff cope with adversity and recover from stressful experiences quickly and effectively to adapt and cope with changeable situations such as crises, workloads, trauma, and other adversities (17). Studies have shown that people with high levels of resilience can easily adapt to setbacks and recover from adverse work environments such as high stress and high load. In the context of the COVID-19 pandemic, resilience can serve as a potential capacity for medical staff to protect themselves (18), helping them effectively manage and deal with stressful situations caused by the pandemic, in order to cope with disasters and survive crises (19). The effort-reward imbalance (ERI) model suggests that medical staff experience job burnout and stress response when the effort invested (e.g., time, effort, and responsibility) does not match the rewards they receive (e.g., pay, respect, and career opportunities) (20–22). When they feel over-committed, they have unrealistic expectations of their work and put in inappropriate effort (23). Due to the constant change between rewards and effort, when over-invested medical staff are in a high-effort and low-reward environment, it will aggravate the occurrence of anxiety, depression, stress, insomnia, and other psychological conditions. Studies have shown that people with high effort and low reward have a significantly higher risk of anxiety and depression than people with low effort and high reward (21, 24).

Studies have shown that medical staff, one of the main groups affected by the pandemic, caring for COVID-19 patients have moderate or high levels of burnout, stress, and anxiety compared to other healthcare Settings (13). In addition, due to the significant increase in the workload of medical staff in the short period of time after policy adjustments, physical exhaustion, high risk of transmission, ethical decision-making issues in the allocation of medical resources, and the effort-reward imbalance may further damage their resilience and exacerbate the impact on their physical and mental health, resulting in stress, anxiety, depression, and insomnia (14). Therefore, it is urgent and necessary to investigate the mental health of medical staff during this period.

Presently, although studies of mental health problems among medical staff during the pandemic have been reported in Wuhan, Shanghai, and other places in China, the survey has focused on local areas and the broader context of outbreak nationwide lock-down, and results vary by time and location (16). Based on the post-epidemic era and the major adjustment of epidemic prevention policy in China, this study investigated the current situation and influencing factors of anxiety, depression, stress, insomnia, resilience, and ERI among medical staff in this period, which can provide a reference for the future routine management of epidemic and intervention measures of large-scale public health emergencies.

## 2. Methods

### 2.1. Study design and participants

The survey was conducted from 6 February to 27 March 2023, based on a non-random sample design. The online questionnaire administered by the web-based survey platform surveyed medical staff working in hospitals during the epidemic. The inclusion criteria are as follows: (a) age range from 18 to 65 years; (b) Medical staff; (c) Voluntary participation in the survey. Exclusion criteria: (a) Have a history of certain psychiatric and physical disorders; (b) Have taken sleep regulation medications; (c) Not engaged in front-line clinical medical work. During the investigation, the researcher explained the research purpose to the respondents and obtained informed consent. Respondents scanned the QR code of the online questionnaire through mobile terminals, logged in, and filled out the questionnaire according to the authorization of their social accounts. Only one answer can be provided by the same IP address, and all items are set as required answers. The questionnaire can only be submitted after all items have been completed. Otherwise, the system automatically records the result as incomplete. The test time was set by the pre-test results, and questionnaires whose filling time was less than 200 s and longer than 1,800 s were deleted. We set confidence coefficient z = 1.96, expected incidence *p* = 0.5, allowable error d = 0.05, 95% confidence interval. By the following formula, we calculated n = 384. Considering a loss factor of 10%, the final sample size was 423.

$$N=Z^{2}\times \frac{P\times \left(1-P\right)}{d^{2}}$$

### 2.2. Outcome measures

#### 2.2.1. Demographics

Social demographic data included gender, profession, age, department, education level, working years, professional title grade, etc. Other questions included whether they had been vaccinated, when and where they were infected, Symptom duration, Rest time after infection, treatment, when they returned to work, and working hours during the pandemic.

#### 2.2.2. Assessment scales

##### 2.2.2.1. Hospital anxiety and depression scale

This scale was developed by Zigmond and Snaith and used to screen for symptoms of non-psychotic anxiety and depression (25). The scale was A 14-item self-rating scale consisting of 2 subscales with 7 items each for anxiety (HDS-A) and depression (HDS-D). Each item was scored at Likert Level 4 (0~3 points), and the score range of each subscale was 0~21 points. The scores were divided into 0 to 7 as asymptomatic. Suspicious symptoms are 8 to 10, and the 11 to 21 range were definite symptoms. The Cronbach’s α of the total volume scale was 0.890, and the Cronbach’s α of the anxiety and depression subscales were 0.820 and 0.807, respectively.

##### 2.2.2.2. Perceived stress scale

The PSS is used to measure the degree of an individual’s perception of stress. This scale has five options for each entry: never, almost never, sometimes, often, and always, with a score of 0 to 4. Among them, items 4, 5, 6, 7, 9, 10, and 13 belong to the negative items (26). Hewitt et al. named this dimension as “perceived coping ability.” Items 1, 2, 3, 8, 11, 12, and 14 belong to the positive items, which is named “perceived distress” (27). The score ranges from 0 to 56 points, with a higher score indicating greater stress, and more than 25 is considered to be excessive stress and in a state of health risk. The scale of Cronbach’s α is 0.954 (28).

##### 2.2.2.3. The effort-reward imbalance

The ERI scale consists of three parts: effort, reward, and over-commitment, with 23 items (29). Six items measured “effort” scores of 6 to 30, 11 items measured “reward” scores of 11 to 55, and 6 items measured “over-commitment” scores of 6 to 24. The ratio is computed by the formula: e/(r*c). “e” defines the score of the effort scale, “r” defines the score of the reward scale, and “c” defines the ratio of the number of effort items and the number of reward items (6/11). In the results, if ERI is greater than 1, it is considered to be the group with high effort and low reward, while ERI less than or equal to 1 is considered to be the group with low effort and high reward, meaning a balance of effort and reward. Those with scores in the top third of the over-commitment factor were considered over-committed. Cronbach’s α of effort, reward, and over-commitment were 0.78, 0.81, and 0.74, respectively (30).

##### 2.2.2.4. Insomnia severity index

This scale was developed by Bastien to assess the severity of individual subjective insomnia (31). A higher score indicates a more severe level of insomnia. The scale consists of 7 items, each of which is scored at level 0 to 4, the total score ranges from 0 to 28 points. Insomnia is considered to exist if more than 7 points are scored, which can be divided into no significant insomnia (0 to 7 points), sub-insomnia (8 to 14 points), clinical insomnia (15 to 21 points), and severe insomnia (>21 points; Cronbach’s α = 0.93) (32).

##### 2.2.2.5. Medical staff resilience scale:

This scale was compiled by Zhu et al. (33) to assess the level of resilience of Chinese medical staff (33). The scale included decision coping (6 items), interpersonal connection (4 items), rational thinking (4 items), and flexible adaptation (4 items), with a total of 18 items in 4 dimensions. All Likert 5 points were scored, and 1–5 points were assigned from completely disagree to completely agree. The total score ranges from 18 to 90, with higher scores indicating higher levels of stress resistance. The Cronbach’s α coefficient of the scale is 0.907, 0.866, 0.797, 0.696, and 0.786 for the four dimensions, respectively.

#### 2.2.3. Statistical analysis

The SPSS 27.0 program was used to statistically analyze the data. Qualitative variables were described by frequency distribution and percentage, while quantitative variables were described by the mean and standard deviation. Independent sample *T*-test and one-way analysis of variance were used. Pearson’s correlation analysis was used to examine associations between anxiety, depression, stress, insomnia, and resilience. Binary logistic regression was used to analyze the relevant influencing factors, and the OR value was calculated. All statistical tests were two-tailed, and a *p*-value of *p* < 0.05 was considered statistically significant.

## 3. Results

### 3.1. Demographic characteristics

A total of 2,530 pieces of data were collected, excluding 142 data with answer times less than 200 s and more than 1,800 s, 283 non-front-line clinical staff, and 67 invalid questionnaires, and finally retained 2,038 data, and the effective rate of questionnaire responses was 80.6%. Female medical staff (79.4%), doctors (52.6%), and nurses (47.4%) account for a relatively balanced proportion and most of them were under 35 years old (61.2%). We found 88.6% of medical staff were infected with the virus during this period, and 48.9% of infections occurred in hospitals. Specific general information and the results of the scales are shown in Table 1.

**Table 1:** Demographic characteristics (*n* = 2,038).

Variables	*N*	(%)		HDS-A	HDS-D	PSS	ISI	Resilience	ERI
Gender			*p*	<0.001^***^	0.342	<0.001^***^	0.007^**^	0.083	0.562
Male	420	20.6		8.22 ± 1.92	7.96 ± 2.10	19.95 ± 7.81	6.55 ± 5.41	77.47 ± 11.67	0.77 ± 0.60
Female	1,618	79.4		8.66 ± 1.90	8.07 ± 2.02	22.54 ± 7.56	7.36 ± 5.51	76.37 ± 10.86	0.75 ± 0.55
Profession			*p*	<0.001^***^	<0.001^***^	<0.001^***^	<0.001^***^	<0.001^***^	<0.001^***^
Doctors	1,071	52.6		8.42 ± 1.90	7.85 ± 2.00	20.68 ± 7.80	6.39 ± 5.12	77.69 ± 10.78	0.71 ± 0.5
Nurses	967	47.4		8.73 ± 1.92	8.26 ± 2.05	23.49 ± 7.27	8.08 ± 5.76	75.38 ± 11.19	0.80 ± 0.62
Age (years)			*p*	0.829	0.756	<0.001^***^	0.157	<0.001^***^	0.638
18–35	1,248	61.2		8.56 ± 1.95	8.07 ± 2.02	22.31 ± 7.52	7.37 ± 5.48	75.87 ± 10.93	0.75 ± 0.54
36–50	747	36.7		8.59 ± 1.87	8.00 ± 2.05	21.78 ± 7.84	6.94 ± 5.53	77.54 ± 11.12	0.77 ± 0.59
51–65	43	2.1		8.42 ± 1.84	8.12 ± 2.28	17.16 ± 7.89	6.40 ± 5.60	81.40 ± 10.28	0.70 ± 0.45
Department			*p*	0.006^**^	0.006^**^	0.003^**^	<0.001^***^	0.004^**^	0.05
Emergency	98	4.8		8.80 ± 1.90	8.53 ± 1.86	22.99 ± 6.35	8.79 ± 5.18	73.78 ± 10.71	0.78 ± 0.53
ICU	58	2.8		9.43 ± 2.33	8.74 ± 2.28	25.29 ± 7.03	9.97 ± 6.41	71.95 ± 12.33	0.91 ± 0.57
Infectious disease	40	2		8.58 ± 1.77	8.45 ± 2.21	20.58 ± 8.36	5.93 ± 5.53	77.93 ± 11.27	0.63 ± 0.28
Respiratory medicine	28	1.4		8.29 ± 2.09	7.71 ± 1.92	18.43 ± 8.76	6.79 ± 6.05	77.57 ± 9.57	0.67 ± 0.26
Clinical laboratory	60	2.9		8.87 ± 2.13	8.37 ± 1.96	21.82 ± 7.63	8.62 ± 4.60	75.68 ± 11.07	0.59 ± 0.28
Radiological department	76	3.7		8.25 ± 1.92	8.07 ± 2.10	22.20 ± 7.62	6.26 ± 5.40	77.29 ± 11.54	0.72 ± 0.60
other	1,678	82.3		8.53 ± 1.88	7.98 ± 2.03	21.93 ± 7.71	7.03 ± 5.46	76.88 ± 10.95	0.76 ± 0.57
Education level			*p*	0.315	0.113	0.003**	0.013^*^	0.085	0.176
Below	11	0.5		7.64 ± 2.11	8.09 ± 2.66	19.27 ± 8.76	5.09 ± 4.99	80.82 ± 12.55	0.57 ± 0.23
Technical secondary school	7	0.3		7.86 ± 2.19	8.14 ± 1.95	20.86 ± 7.65	7.14 ± 4.91	78.43 ± 11.93	0.40 ± 0.19
Junior	114	5.6		8.44 ± 1.79	8.34 ± 2.20	21.93 ± 7.41	7.03 ± 5.26	78.97 ± 10.28	0.68 ± 0.58
Bachelor	1,426	70		8.59 ± 1.90	8.09 ± 2.02	22.45 ± 7.47	7.47 ± 5.62	76.58 ± 11.24	0.77 ± 0.59
Master	410	20.1		8.59 ± 1.97	7.89 ± 2.07	20.80 ± 8.26	6.40 ± 5.19	76.18 ± 10.59	0.73 ± 0.43
Doctor	70	3.4		8.29 ± 1.99	7.60 ± 1.75	20.84 ± 8.02	6.69 ± 4.91	74.74 ± 9.81	0.80 ± 0.58
Working years			*p*	0.003^**^	<0.001^***^	<0.001^***^	<0.001^***^	0.522	<0.001^***^
<3	311	15.3		8.20 ± 1.84	7.64 ± 1.82	19.69 ± 7.53	5.94 ± 4.93	76.33 ± 10.33	0.64 ± 0.40
3–5	267	13.1		8.52 ± 2.11	8.03 ± 2.04	22.03 ± 7.72	6.80 ± 5.41	76.26 ± 11.05	0.77 ± 0.58
5–10	511	25.1		8.66 ± 1.91	8.22 ± 2.09	23.12 ± 7.41	7.79 ± 5.50	76.21 ± 11.06	0.76 ± 0.50
>10	949	46.6		8.65 ± 1.87	8.09 ± 2.06	22.17 ± 7.71	7.39 ± 5.64	76.99 ± 11.24	0.79 ± 0.62
Professional title grade			*p*	0.153	0.063	<0.001^***^	0.631	0.433	0.056
Junior	1,007	49.4		8.51 ± 1.92	8.12 ± 2.03	22.13 ± 7.40	7.26 ± 5.46	76.92 ± 10.94	0.72 ± 0.54
Middle	853	41.9		8.67 ± 1.93	8.04 ± 2.04	22.35 ± 7.75	7.20 ± 5.54	76.2 ± 11.17	0.77 ± 0.57
Sub-Senior	166	8.1		8.37 ± 1.75	7.67 ± 2.02	19.68 ± 8.62	6.87 ± 5.66	76.51 ± 11.00	0.84 ± 0.62
Senior	12	0.6		8.83 ± 2.52	7.67 ± 1.97	20.42 ± 7.87	5.58 ± 4.48	79.33 ± 9.21	0.73 ± 0.37
Vaccine injection			*p*	0.563	0.901	0.843	0.682	0.559	0.981
Yes	2,008	98.5		8.56 ± 1.91	8.05 ± 2.04	22.01 ± 7.68	7.19 ± 5.49	76.58 ± 11.03	0.75 ± 0.56
No	30	1.5		8.77 ± 1.99	8.00 ± 1.58	21.73 ± 7.88	7.60 ± 6.12	77.77 ± 11.49	0.76 ± 0.78
Whether infected or not			*p*	0.046^*^	0.108	0.103	0.02*	0.091	0.099
Yes	1,810	88.8		8.59 ± 1.93	8.07 ± 2.04	22.11 ± 7.64	7.29 ± 5.54	76.45 ± 11.05	0.76 ± 0.57
No	228	11.2		8.34 ± 1.77	7.84 ± 1.98	21.23 ± 7.93	6.39 ± 5.11	77.76 ± 10.84	0.70 ± 0.45
Location of infection			*p*	0.437	0.403	0.472	0.145	0.023^*^	0.366
Uninfected	228	11.2		8.34 ± 1.77	7.84 ± 1.98	21.23 ± 7.93	6.39 ± 5.11	77.76 ± 10.84	0.70 ± 0.45
Department	818	40.1		8.61 ± 1.94	8.10 ± 1.94	21.97 ± 7.43	7.25 ± 5.50	75.86 ± 10.80	0.75 ± 0.53
Other parts of the hospital	177	8.7		8.54 ± 1.95	8.01 ± 2.06	21.98 ± 8.36	7.74 ± 5.74	78.18 ± 10.85	0.74 ± 0.48
At home	764	37.5		8.60 ± 1.92	8.08 ± 2.15	22.31 ± 7.61	7.26 ± 5.51	76.79 ± 11.25	0.77 ± 0.63
Outdoors	51	2.5		8.51 ± 1.87	7.76 ± 1.92	21.84 ± 9.10	7.04 ± 5.96	74.84 ± 12.12	0.83 ± 0.64
Symptom duration			*p*	<0.001^***^	0.322	0.004^**^	<0.001^***^	0.219	0.068
Uninfected	228	11.2		8.34 ± 1.77	7.84 ± 1.98	21.23 ± 7.93	6.39 ± 5.11	77.76 ± 10.84	0.70 ± 0.45
<3 days	608	29.8		8.37 ± 1.83	7.99 ± 2.09	21.31 ± 7.72	6.63 ± 5.36	77.01 ± 11.02	0.79 ± 0.65
3–5 days	658	32.3		8.67 ± 1.94	8.16 ± 2.05	22.76 ± 7.55	7.37 ± 5.42	76.04 ± 11.06	0.73 ± 0.50
5–7 days	329	16.1		8.65 ± 1.93	8.06 ± 1.95	21.87 ± 7.82	7.98 ± 5.84	76.11 ± 11.48	0.73 ± 0.53
>7 days	215	10.5		8.91 ± 2.12	8.06 ± 2.02	22.72 ± 7.25	7.89 ± 5.78	76.66 ± 10.45	0.82 ± 0.60
Rest time after infection			*p*	0.007^**^	0.284	0.023*	0.035^*^	0.152	0.336
Uninfected	228	11.2		8.34 ± 1.77	7.84 ± 1.98	21.23 ± 7.93	6.39 ± 5.11	77.76 ± 10.84	0.7 ± 0.45
<3 days	616	30.2		8.41 ± 1.91	8.00 ± 1.95	21.65 ± 7.83	7.10 ± 5.63	76.96 ± 11.34	0.75 ± 0.56
3–5 days	467	22.9		8.60 ± 1.94	8.13 ± 2.03	21.87 ± 7.60	7.01 ± 5.42	76.45 ± 11.03	0.75 ± 0.62
5–7 days	460	22.6		8.74 ± 1.86	8.03 ± 2.08	22.31 ± 7.59	7.68 ± 5.55	75.65 ± 10.62	0.78 ± 0.57
>7 days	267	13.1		8.76 ± 2.04	8.21 ± 2.19	23.23 ± 7.31	7.56 ± 5.51	76.67 ± 11.15	0.79 ± 0.52
Treatment			*p*	<0.001^***^	0.241	0.015*	<0.001^***^	0.182	0.004^**^
Uninfected	228	11.2		8.34 ± 1.77	7.84 ± 1.98	21.23 ± 7.93	6.39 ± 5.11	77.76 ± 10.84	0.70 ± 0.45
Self-healing at home	1,626	79.8		8.65 ± 1.93	8.09 ± 2.04	22.27 ± 7.57	7.35 ± 5.52	76.36 ± 11.03	0.76 ± 0.58
Hospitalization	10	0.5		8.40 ± 2.22	8.10 ± 2.38	23.20 ± 6.75	12.80 ± 5.77	80.40 ± 10.15	1.27 ± 1.36
Untreated	174	8.5		8.05 ± 1.82	7.89 ± 2.09	20.57 ± 8.27	6.48 ± 5.51	77.10 ± 11.31	0.69 ± 0.42
Time of return to work			*p*	0.268	0.592	0.02*	0.007^**^	0.007^**^	<0.001^***^
Uninfected	228	11.2		8.34 ± 1.77	7.84 ± 1.98	21.23 ± 7.93	6.39 ± 5.11	77.76 ± 10.84	0.70 ± 0.45
Return to work with symptoms	739	36.3		8.60 ± 1.97	8.08 ± 1.97	22.18 ± 7.66	7.63 ± 5.74	75.41 ± 11.09	0.79 ± 0.61
1–3 days after recovery	600	29.4		8.55 ± 1.90	8.08 ± 2.02	21.60 ± 7.41	6.77 ± 5.14	77.23 ± 10.94	0.69 ± 0.46
3–7 days after recovery	244	12		8.56 ± 1.86	8.07 ± 2.23	21.94 ± 7.88	7.47 ± 5.82	77.15 ± 10.69	0.76 ± 0.55
>7 days after recovery	227	11.1		8.74 ± 1.96	8.01 ± 2.14	23.38 ± 7.85	7.39 ± 5.53	77.02 ± 11.41	0.85 ± 0.71
Working hours			*p*	0.035^*^	0.022^*^	0.055	<0.001^***^	0.015^*^	<0.001^***^
<8 h	577	28.3		8.41 ± 1.87	7.83 ± 1.98	21.48 ± 7.76	6.44 ± 5.23	77.71 ± 10.79	0.68 ± 0.48
8–10 h	905	44.4		8.59 ± 1.89	8.13 ± 2.02	22.24 ± 7.35	7.29 ± 5.45	76.44 ± 11.13	0.75 ± 0.55
11–12 h	107	5.3		8.95 ± 2.07	8.28 ± 2.18	23.48 ± 8.03	7.82 ± 5.39	74.92 ± 11.46	0.87 ± 0.63
>12 h	449	22		8.61 ± 1.97	8.10 ± 2.09	21.87 ± 8.09	7.81 ± 5.86	75.89 ± 10.94	0.82 ± 0.64

^*^p < 0.05, ^**^p < 0.01, ^***^p < 0.001.

### 3.2. The proportion of anxiety, depression, insomnia, stress, and ERI

In the evaluation of anxiety, depression, insomnia, 68.5% and 53.9% of the medical staff had different degrees of anxiety and depression, and 43.2% and 40.2% of the medical staff had insomnia and excessive stress. In the measurement of the ERI, high-effort and low-reward accounted for 14.2%, and low-effort and high-reward accounted for 85.8% (see Table 2).

**Table 2:** The proportion of anxiety, depression, insomnia, stress and ERI.

	Anxiety	Depression	Insomnia		Stress		ERI			
Asymptomatic	642 (31.5%)	940 (46.1%)	No significant insomnia	1,158 (56.8%)	Excessive stress	820 (40.2%)	High-effort and low-reward	290 (14.2%)	Not over-commitment	1,092 (53.6%)
Mild	1,078 (52.9%)	843 (41.4%)	Sub insomnia	677 (33.2%)						
Moderate	312 (15.3%)	248 (12.2%)	Clinical insomnia	188 (9.2%)	No stress	1,218 (59.8%)	Low-effort and high-reward	1,748 (85.8%)	Over-commitment	946 (46.4%)
Severe	6 (0.3%)	7 (0.3%)	Severe insomnia	15 (0.7%)						

### 3.3. Analysis of factors related to anxiety, depression, insomnia, stress, and ERI

In this study, women were risk factors for anxiety (OR = 1.360, *p* < 0.05) and stress (OR = 1.334, *p* < 0.05). Higher age was a protective factor for insomnia (OR = 0.709, *p* < 0.01) and stress (OR = 0.760, *p* < 0.05). Nurses (OR = 1.298, *p* < 0.05), higher education (OR = 1.242, *p* < 0.05), and long working years (OR = 1.247, *p* < 0.001) were risk factors for anxiety. To the contrary, high professional title grade was a protective factor for anxiety (OR = 0.831, *p* < 0.05) and depression (OR = 0.750, *p* < 0.001). Being a nurse, longer working life, and longer working hours during the pandemic are risk factors for depression, insomnia, and stress. Non-infection with COVID-19 (OR = 0.486, *p* < 0.05) and prolonged symptoms of COVID-19 (OR = 1.152, *p* < 0.01) were protective and risk factors for insomnia, respectively. In addition, this study found that nurses (OR = 1.465, *p* < 0.05), higher education (OR = 1.476, *p* < 0.001), and longer working hours were more likely to show the ERI (OR = 1.312, *p* < 0.001; see Table 3).

**Table 3:** Analysis of factors related to anxiety, depression, insomnia, stress, and ERI.

	Anxiety		Depression		Insomnia		Stress		ERI	
	OR (95%CI)	*P*	OR (95%CI)	*P*	OR (95%CI)	*P*	OR (95%CI)	*P*	OR (95%CI)	*P*
Gender	1.360 (1.066,1.735)	0.013^*^	0.847 (0.669,1.073)	0.168	1.039 (0.816,1.323)	0.758	1.334 (1.037,1.718)	0.025*	0.760 (0.547,1.057)	0.103
Profession	1.298 (1.039,1.620)	0.021^*^	1.391 (1.131,1.712)	0.002^**^	1.397 (1.131,1.725)	0.002^**^	1.738 (1.404,2.151)	<0.001^***^	1.465 (1.066,2.012)	0.019*
Age	0.905 (0.716,1.143)	0.402	0.926 (0.745,1.151)	0.488	0.709 (0.568,0.886)	0.002^**^	0.760 (0.607,0.951)	0.017^*^	0.786 (0.571,1.083)	0.141
Education level	1.242 (1.042,1.480)	0.016^*^	1.063 (0.899,1.257)	0.475	1.156 (0.968,1.381)	0.109	1.146 (0.953,1.379)	0.147	1.476 (1.133,1.924)	0.004^**^
Working years	1.247 (1.110,1.400)	<0.001^***^	1.244 (1.114,1.388)	<0.001^***^	1.278 (1.141,1.432)	<0.001^***^	1.333 (1.187,1.498)	<0.001^***^	1.104 (0.939,1.299)	0.230
Professional title grade	0.831 (0.692,0.998)	0.047^*^	0.750 (0.631,0.891)	<0.001^***^	0.865 (0.725,1.031)	0.106	0.860 (0.718,1.030)	0.101	1.230 (0.965,1.567)	0.094
Whether infected or not	0.591 (0.291,1.200)	0.146	1.143 (0.592,2.207)	0.691	0.486 (0.249,0.947)	0.034^*^	0.823 (0.464,1.462)	0.507	0.850 (0.332,2.181)	0.736
Symptom duration	1.103 (0.992,1.227)	0.069	1.042 (0.945,1.148)	0.414	1.152 (1.044,1.270)	0.005^**^	1.075 (0.976,1.184)	0.141	1.031 (0.899,1.181)	0.665
Working hours	1.048 (0.957,1.147)	0.309	1.112 (1.022,1.211)	0.014^*^	1.159 (1.064,1.262)	<0.001^***^	1.112 (1.021,1.212)	0.015^*^	1.312 (1.170,1.471)	<0.001^***^

Age, education level, working years, professional title grade, and working hours during the epidemic period are regarded as continuous variables. Doctors: 0, nurses: 1; Uninfected: 0, infected: 1. ^*^p < 0.05，^**^p < 0.01, ^***^p < 0.001.

### 3.4. Correlation analysis of HAD, PSS-14, ISI, resilience, and ERI

Pearson’s correlation analysis found that there was a certain correlation between HAD, PSS-14, ERI, ISI, and the results of the stress assessment scale. Among them, anxiety, depression, stress, insomnia, and ERI are positively correlated (*p* < 0.001), while resilience is negatively correlated with anxiety, depression, stress, insomnia, and ERI (*p* < 0.001; see Table 4).

**Table 4:** Correlation analysis of HAD, PSS-14, ISI, resilience and ERI.

	Mean	SD	Anxiety	Depression	Stress	Insomnia	Resilience	ERI
Anxiety	8.57	1.91	1	0.288^***^	0.495^***^	0.374^***^	−0.271^***^	0.345^***^
Depression	8.05	2.04	0.288^***^	1	0.365^***^	0.307^***^	−0.188^***^	0.216^***^
Stress	22.01	7.68	0.495^***^	0.365^***^	1	0.484^***^	−0.414^***^	0.444^***^
Insomnia	7.19	5.50	0.374^***^	0.307^***^	0.484^***^	1	−0.325^***^	0.411^***^
Resilience	76.60	11.03	−0.271^***^	−0.188^***^	−0.414^***^	−0.325^***^	1	−0.388^***^
ERI	0.75	0.56	0.345^***^	0.216^***^	0.444^***^	0.411^***^	−0.388^***^	1

Pearson’s correlation analysis, ^***^p < 0.001.

### 3.5. Effects of ERI on HAD, PSS-14, ISI, and resilience

With ERI results as grouping variables and scores of HAD, PSS-14, ISI, and resilience as test variables, an independent sample *T*-test was conducted. It was found that the high-effort and low-reward group and the over-commitment group had higher scores of anxiety, depression, stress, and insomnia and lower scores of resilience and its dimensions than the low-effort and high-reward group and the no over-commitment group, respectively. The difference was statistically significant (*p* < 0.001; see Table 5).

**Table 5:** Effects of ERI on HAD, PSS-14, ISI, and resilience.

ERI	Anxiety	Depression	Perceived coping ability	Perceived distress	Stress	Insomnia	Decision coping	Interpersonal connection	Rational thinking	Flexible adaptation	Resilience
High-effort and low-reward	9.44 ± 2.17	8.66 ± 2.31	13.2 ± 4.92	13.59 ± 4.88	26.78 ± 6.78	10.91 ± 6.09	24.12 ± 4.03	16.08 ± 2.78	15.54 ± 2.97	14.97 ± 3.26	70.71 ± 11.17
Low-effort and high-reward	8.42 ± 1.83	7.94 ± 1.97	11.33 ± 5.43	9.89 ± 4.36	21.22 ± 7.53	6.57 ± 5.15	25.95 ± 3.77	17.58 ± 2.41	17.07 ± 2.68	16.97 ± 2.71	77.58 ± 10.7
T	7.616	5.006	5.496	13.139	12.735	11.471	−7.596	−9.603	−8.859	−9.913	−10.057
P	<0.001^***^	<0.001^***^	<0.001^***^	<0.001^***^	<0.001^***^	<0.001^***^	<0.001^***^	<0.001^***^	<0.001^***^	<0.001^***^	<0.001^***^
Not over-commitment	8.14 ± 1.78	7.72 ± 1.89	10.62 ± 5.5	9.11 ± 4.32	19.73 ± 7.62	5.59 ± 4.85	26.07 ± 3.76	17.78 ± 2.37	17.27 ± 2.66	17.28 ± 2.63	78.39 ± 10.55
Over-commitment	9.06 ± 1.94	8.42 ± 2.13	12.72 ± 5.04	11.92 ± 4.51	24.64 ± 6.87	9.04 ± 5.63	25.25 ± 3.93	16.9 ± 2.61	16.37 ± 2.82	16 ± 3.01	74.53 ± 11.22
T	−11.207	−7.755	−8.925	−14.332	−15.275	−14.667	4.774	7.975	7.362	10.224	8.009
P	<0.001^***^	<0.001^***^	<0.001^***^	<0.001^***^	<0.001^***^	<0.001^***^	<0.001^***^	<0.001^***^	<0.001^***^	<0.001^***^	<0.001^***^

*** *p* < 0.001.

## 4. Discussion

Within a short period of time after the epidemic policy was opened, more than 50% of medical staff suffered from anxiety and depression to varying degrees, and more than 40% of medical staff suffered from excessive stress and insomnia. At the same time, the investigation showed that there was a certain correlation between the five scales of HAD, PSS-14, ERI, ISI, and resilience, and the relevant influencing factors mainly included gender, profession, education level, work years, professional title grade, working hours during the epidemic period, etc. The study also found that ERI has some effect on anxiety, depression, stress, insomnia severity, and resilience.

Anxiety, depression, and insomnia among medical staff found in this study accounted for 42% of anxiety and 33% of depressive symptoms reported in Aymerich et al. ‘s meta-analysis (34). Plus, Lai et al. reported that the prevalence rates of anxiety, depression, and insomnia among medical staff were 44.6%, 50.4%, and 34.0%, respectively (35). The reason may be that there has never been a large-scale outbreak nationwide in China’s epidemic prevention process in the past, and the major adjustment and opening of the epidemic policy this time is nationwide and timely. However, given China’s huge population base and limited medical resources, this undoubtedly brings a heavy burden on hospitals and medical staff in a short period of time. In addition, the study found that infection and symptom duration were risk factors for insomnia, with nearly 80% of infected medical staff self-medicating at home to treat and relieve symptoms. However, after nearly 50% of medical workers were infected with COVID-19 in hospital, more than 35% of them stayed on their posts to ensure the normal operation of hospitals to treat patients, and nearly 30% were back to work 3 days after infection. It may also be one of the important causes of their anxiety and insomnia. Working hours are also a risk factor for depression, insomnia, and stress, with more than 70 percent of medical staff working more than 8 h a day and 22 percent working more than 12 h a day. Previous evidence has also shown that the overwhelming workload pressure in the COVID-19 pandemic can cause great psychological stress and mental disorders for medical staff, especially increased anxiety responses, depressive symptoms, and stress, which is consistent with the findings of this study (36).

In this study, we found that during the COVID-19 pandemic, women were more likely to experience anxiety, depression, stress, and insomnia than men. Nurses were more likely to experience stress than doctors, which is consistent with the results of previous studies (37, 38). At the same time, it was found that compared with doctors, nurses had lower resistance and scores of all dimensions, and were more likely to have ERI. This may be due to the fact that nurses, most of whom are female, are directly responsible for nucleic acid collection and care of COVID-19 patients (39), face a greater risk of infection and workload, but are lower than doctors in terms of professional identity, social status, and salary (40), thus making them more prone to psychological symptoms and ERI. Therefore, more mental health support, social support, and well-being need to be provided to nurses during the response to major public health events (18, 41).

Unlike previous studies, high education level was found to be a risk factor for anxiety and the ERI in this study, but not a protective factor for insomnia (42). The reason may be that the public already has a clear understanding of COVID-19, and the awareness of COVID-19 varies according to different levels of education, so anxiety, depression, insomnia, and other conditions caused by it are not the main reason. The reason is more likely to be that the higher well-being expected by highly educated medical staff has not been achieved, because the ERI only led to anxiety, and the level of psychological disorders has not yet led to depression and insomnia. In regression analysis, we found that advanced age was a protective factor for stress and insomnia, and higher professional title grade was a protective factor for anxiety and depression, as found by Zhou and Irene Teo et al. (43, 44). Because anxiety is highly associated with burnout, older and higher professional title-grade medical staff tended to have more experience and lower levels of burnout, they are more adept at coping with emergencies than younger medical staff, and they have even participated in the prevention and control of SARS (44–46). At the same time, working years were found to be a risk factor for anxiety, depression, stress, and insomnia. A long working life does not mean a match for a higher professional title grade, and medical workers with higher working years are more likely to suffer from anxiety, pressure, and other psychological disorders due to job burnout when dealing with the high workload of the epidemic (47).

Further analysis showed that medical staff working in departments that were in close contact with COVID-19 patients, such as intensive care units, emergency departments, respiratory departments, and infectious disease departments, showed more psychological disorders such as anxiety, depression, and stress than clinical staff in other departments, consistent with the results of Lu et al. (48). In addition, due to the need to monitor a large number of nucleic acid specimens, compared with other departments above, the laboratory department has more prominent problems in insomnia and ERI.

In the correlation analysis, there was a certain correlation between the five scales; anxiety, depression, stress, insomnia, and the ERI were all positively correlated, while stress resistance was negatively correlated with anxiety, depression, stress, insomnia, and ERI. Under the influence of long-term negative events, people tend to be more vulnerable, and medical staff with higher stress levels tend to suffer from anxiety, depression, insomnia, and other symptoms (16, 49). Consistent with previous studies, high resilience means that individuals are more likely to bounce back and adapt quickly to adversity (50, 51). People with higher resilience are more likely to proactively cope with setbacks or difficulties, have greater resilience to stress, greater social adjustment, and have higher levels of self-evaluation and self-confidence (52). Therefore, resilience can not only serve as an important buffer against psychological trauma, stress, insomnia, and psychological distress. Individuals’ mental resilience can also be assessed to predict mental health status, stress perception, and sleep quality (19). However, it is worth noting that the results of a comprehensive review showed that while medical staff exhibited moderate levels of resilience on average during the COVID-19 pandemic, they were not immune to the negative psychological effects of working during the pandemic (53). Therefore, how to maintain and improve the resilience of medical staff to cope with large-scale public health emergencies needs further study.

This study also found that when the ERI occurred among medical staff, the high effort and low reward group and the over-commitment group were more prone to anxiety, depression, stress, and insomnia than the low-effort and high-reward group and the non-over-commitment group, respectively. Nursing, higher education level, and working hours are risk factors for the ERI. Previous studies have shown that a lack of reciprocity between the time and effort individuals invest in their work and the money, respect, recognition, and career opportunities they receive can lead to negative emotions, which in turn affect the autonomic nervous system’s sustained stress response, leading to insomnia and mental health problems (21, 22, 54). At present, many studies have confirmed that ERI is positively correlated with adverse mental state and perceived stress, and people in situations of high effort and low reward have more adverse physical health conditions and mental health problems (54), which is consistent with the results of this study. Therefore, this study believes that, when dealing with major public health emergencies, improving the social support and well-being of medical staff and improving the imbalance of reward may effectively improve their resilience and reduce their anxiety, depression, and other psychological disorders, stress, insomnia, and other symptoms. On the other hand, attention should also be paid to the working hours and the contribution of nurses and highly educated medical staff in responding to such incidents. Because in the long run, nurses and highly educated medical staff are under pressure to work long hours, exert greater effort for lower reward, and may use self-protection strategies to distance themselves emotionally, which in turn negatively affects the quality of medicine and care (22). We need to focus not only on the mental health and well-being needs of medical staff from an individual perspective. It is also necessary to rationally allocate medical and health human resources at the organizational level, reduce the overworked state of medical staff, design scientific and reasonable salary systems and performance evaluation indicators, and reflect the value of technical labor of medical staff. In addition, we also need to continuously optimize and improve the evaluation effectiveness of psychological, stress, sleep, burnout, and other scales, and try to use new procedures and tools to detect the psychological condition of medical staff more timely and accurately, because prevention and remission are far more important than cure (55–57).

## 5. Conclusion

This study found that anxiety, depression, stress, insomnia, and other conditions of medical staff in China during the period of policy opening to COVID-19 were higher than those in previous studies. Women, nurses, higher education level, more working years and working hours, high effort and low reward are risk factors for anxiety, depression, stress, and insomnia. Attention should be paid to the influence of the above factors on the psychological status of medical staff. There are positive correlations among anxiety, depression, stress, insomnia, and ERI, and they affect each other. Resilience acts as an important buffer for psychological trauma, stress, insomnia, and the ERI, and can be used to predict and improve the mental health status, stress perception, and sleep quality of medical personnel. This study calls for improving the well-being of medical staff, optimizing the allocation of human resources, focusing on prevention and mitigation, and maintaining the mental health and sleep quality of medical staff in response to major public health emergencies at the individual and organizational levels, so as to promote the improvement of the quality of medicine and care.

### 5.1. Limitations

Some limitations of this study should be noted. First, this study is a single-center study with a non-probability sample survey rather than a random sample survey. In addition, the survey was conducted online rather than face-to-face. Despite these limitations, this study is very important for understanding the psychological status of medical staff in response to major public health emergencies in the future and can provide some reference value for them to formulate psychological care strategies and related epidemic prevention policies.

## Data availability statement

The raw data supporting the conclusions of this article will be made available by the authors, without undue reservation.

## Ethics statement

Ethical review and approval was not required for the study on human participants in accordance with the local legislation and institutional requirements. The study was conducted in accordance with the World Medical Association Code of Ethics (Declaration of Helsinki) with the informed consent of all participants.

## Author contributions

ZhC: study design, data collection, interpretation, analysis, and drafted manuscript. YT: data collection, interpretation, analysis, and revision. TL: investigation, study coordination, and manuscript revision. SH: investigation, data collection, and data analysis. YC: investigation and data collection. LS: project administration, study design, and manuscript revision. ZoC: guarantor of integrity of the entire study, study design, and manuscript revision. All authors contributed to the article and approved the submitted version.

## Funding

The research received funding from the Army Military Medical University Humanities and Social Science foundation project (grant no. 2022XRW15). The funding sources were independent and did not influence the study design, data collection and analysis, study conclusions, and publishing decisions.

## Conflict of interest

The authors declare that the research was conducted in the absence of any commercial or financial relationships that could be construed as a potential conflict of interest.

## Publisher’s note

All claims expressed in this article are solely those of the authors and do not necessarily represent those of their affiliated organizations, or those of the publisher, the editors and the reviewers. Any product that may be evaluated in this article, or claim that may be made by its manufacturer, is not guaranteed or endorsed by the publisher.
